# Addressing Gaps in Competency-Based Medical Education in EEG Instruction, Virtual Learning, and High-Quality Peer Review in Medical Education

**DOI:** 10.1212/NE9.0000000000200107

**Published:** 2023-12-01

**Authors:** Roy E. Strowd

**Affiliations:** From the Department of Neurology, Wake Forest School of Medicine, Winston Salem, NC.

In this December issue of *Neurology*® *Education*, readers will find plenty of great reads to keep them warm next to the winter fire. Several themes emerge from the articles in this issue. First, competency-based medical education (CBME) is a recurring gap identified throughout medical education including in articles on EEG education, neuroradiology instruction, and end-of-rotation comments. CBME is an outcome-based approach that requires a robust assessment system of learner achievement.^1^ In this issue, multiple author groups tackle this challenge by developing standardized EEG assessment examinations. Interested readers can learn from the approaches taken to instrument development by these authors. Second, virtual learning continues to be a common topic in submissions to *Neurology: Education*. This issue highlights ongoing priority areas in virtual learning including one institution's experience with residency second look and another institution's use of a virtual patient management conference to create a zone of proximal development as a theoretical underpinning of this intervention in epilepsy case conference. Finally, this issue includes 2 must-read review articles on gamification in neuroscience and how to conduct a high-quality peer review in medical education.

## Teaching and Assessing Competence in EEG Education

The first group of articles in this issue focus on new approaches to teaching and assessing EEG reading skills. EEG remains a foundational procedural skill for the neurology trainee and is influential in the diagnosis, monitoring, risk stratification, and inpatient management of patients with epilepsy. Despite Hans Berger's first recorded human EEG tracings in the 1920s,^2^ standardized assessment tools that optimally assess EEG reading skills have not been broadly validated. Two articles by Moeller et al.^3^ and Nascimento et al.^4^ take different approaches to tackling this educational gap.

Moeller et al. describe how they developed a nationwide Epilepsy Fellowship In-service Training Examination (EpiFITE). Mirroring the goals of the Residency In-service Training Examination, the authors describe how they developed the examination for epilepsy fellows. The article walks through the validation process describing 4 key validity arguments for EpiFITE: content validity, internal structure, response process validity, and consequential validity. Nascimento et al. take a different approach using an online method for developing and disseminating an EEG multiple-choice examination. Online delivery allowed for widespread dissemination, and authors were able to compare performance across residents, fellows, neurophysiologists, and even non-neurologists and EEG technologists. How much EEG training is needed to read like an expert? These authors suggest at least 8 weeks. In their study, at least 8 weeks of EEG training was needed to perform EEG reading like expert neurophysiologists. Of interest, confidence in recognizing an EEG finding lagged behind performance and took much more than 8 weeks.

Both articles demonstrate the importance of starting examination development with a clear examination blueprint. These articles describe key steps in examination development including beginning with content validity, employing an expert team for item development and quality control, the types of psychometric assessments needed for a new instrument, and the value of consequential validity. Readers gain a great deal from not only knowing about these 2 specific assessment examinations but even more how to go about developing their own validated instrument and the validity arguments needed.

If these 2 articles cover the assessment of EEG reading skills, the remaining articles in this group tackle what is the best approach to learning how to read an EEG? Fernandez et al.^5^ describe a multi-institution effort through the American Epilepsy Society to deliver both synchronous and asynchronous EEG instruction. Although the asynchronous instruction was more widely available, fewer learners completed it. The synchronous sessions were more effective, but participation was lower and limited the strength of the author conclusions. The final study by Pecha et al.,^6^ explores an alternative approach. The authors describe how a virtual patient management conference (i.e., epilepsy surgery conference) improved the learning for trainees. How was this achieved and what does it tell the field about how to teach EEG? The authors suggest that polling conducted in the virtual patient conference helped to tap into the social context of learning. Drawing on the zone of proximal development theory, the authors suggest that the conversations during the conference helped learners reach the edge of their learning and navigate this using interactive polls, faculty discussion, targeted questions, and group interaction that was facilitated by the virtual platform used.^7^

How close are we to achieving a competency-based framework for EEG education? There is still much to be done. However, there are important conclusions from these studies. First, none of these data challenge traditional approaches to teaching EEG reading skills using repetition, mentorship, foundational reading, and a logical sequence when reviewing the EEG. The articles do suggest that foundational learning is an important first step in accuracy and confidence in reading EEG. This may require at least 8 weeks of EEG rotation after which minimum competence in EEG findings (i.e., epileptiform discharges, normal variants, etc.) can be assessed. However, additional practice is needed and provides an opportunity for trainees to gain confidence and efficiency that may be best facilitated by learning with colleagues. Case-based vignettes and patient management conferences may be a key ingredient to creating this social context.

EEG training is only the tip of the iceberg in the procedural skills expected to be mastered by neurology trainees. In their article, Benea et al.^8^ evaluated the competence and readiness of Canadian neurology trainees to interpret various imaging studies for patients with neurologic disease. Although program directors more than residents were confident in the residents' ability, both agreed that there was a lack of clarity of what it takes to be competent to read complex imaging studies reliably.

The last article in this series by Eugene et al.^9^ describes the results of a qualitative study exploring faculty perspectives on end-of-rotation assessments. A summative assessment is a key component of determining learner competence. This article highlights some of the ongoing challenges. Faculty report a lack of training in summative narrative assessment, need examples of text to draw upon, and fear that comments could negatively affects students or lead to retaliation.

## Personalizing the Learning Experience While Maintaining Equity and Inclusion

The middle 2 articles in this issue by Peyton et al.^10^ and Mauricio et al.^11^ address different aspects of personalizing the learner experience. Peyton et al. evaluated learning preferences for medical students rotating through the neurology clerkship. The authors used the learning preference inventory to evaluate both state-based preferences (e.g., learning preferences that are more environmentally dependent and modifiable) and trait-based preferences (e.g., preferences that are more engrained and less malleable over time). As students progressed through their training, a greater preference for individual learning was observed compared with group learning. Overall, concrete delivery of information more than abstract delivery was preferred for all students. However, abstract learners reported greater satisfaction with online and virtual case instruction. These data highlight that one size does not fit all students and selection is of value to learners. This study does not address whether differences in learning preference influence student performance and achievement.

Virtual interviews have become as pervasive as virtual learning in the coronavirus disease 2019 pandemic era. Mauricio et al. describe an experience with a virtual second look program for medical students and describe how this was used to tailor the experience of prospective candidates. Virtual interviewing has reduced travel burdens, financial demands, and improved equity for residency applicants. However, virtual interviews can fall short in providing a clear window into the program, the city, and the culture of a residency. The virtual second look described by Mauricio et al. was not for everyone, with only 22% of applicants attending. However, those who attended reported that the visit influenced their rank list and residency decision.

It seems that virtual learning and interviewing is here to stay and offers enhanced standardization, equity, and flexibility. However, moving forward, in teaching, assessment, and interviewing, it will be critical for educators to ensure that safeguards are in place to protect prospective candidates, that clinical learning occurs at the bedside with a patient, and that assessment ensures that competence to practice is achieved.

## Two Must-Read Review Articles on Gamification and How to Peer Review

The final 2 articles in this issue are reviews that are must-read articles for *Neurology: Education* readers.

Games are fun. They can motivate learners, increase engagement, invite (friendly) competition, create a social context for learning, facilitate critical thinking, help learners construct new knowledge, and even be a gateway for further learning. How do educational games work? What is the neuroscience behind a game that teaches? How do game makers balance edutainment and education in constructing and developing a game? Edwards et al.^12^ address all of these questions in a key review article that discusses the role of gamification in neuroscience and how to create a serious educational game that enhances learning outcomes.

The last article is one that I hope all *Neurology: Education* readers will bookmark. This article reviews an approach to peer reviewing in medical education. Peer review is an essential part of scientific publishing which not only serves to filter the scientific literature but also to improve the quality of work that is disseminated. Over the past year and a half, *Neurology: Education* has received tremendous interest from individuals interested in peer reviewing for the journal. Peer reviewing requires content expertise, critical appraisal skills, the ability to communicate with authors and editors, and an understanding of publication science. Many reviewers have asked for guidance and training in how to conduct a quality peer review in medical education. A group of experts including the inaugural trainee editorial board members of *Neurology: Education* have taken on the task of answering this question. These authors provide a step-by-step approach to how to conduct a high-quality peer review.^13^

Enjoy this issue of *Neurology: Education*. Happy reading.
